# Supplementary material for: Spatially resolved proteomics via tissue expansion
Source: Nat Commun. 2022 Nov 30;13:7242. doi: 10.1038/s41467-022-34824-2 (PMC9712279; doi:10.1038/s41467-022-34824-2)
Supplement: Supplementary file 1 — Supplementary Information [file 41467_2022_34824_MOESM1_ESM.pdf]

**Supplementary Information**

# Spatially Resolved Proteomics via Tissue Expansion

Lu Li[1,2,3,4,5#], Cuiji Sun[1,2,4#], Yaoting Sun[1,2,3,4#], Zhen Dong[1,2,3,4#], Runxin Wu[1,2,3,4,6], Xiaoting Sun[1,2,4], Hanbin Zhang[1,2,4], Wenhao Jiang[1,2,3,4], Yan Zhou[1,2,3,4], Xufeng Cen[7], Shang Cai[1,2], Hongguang Xia[7,8,9], Yi Zhu[1,2,3,4], Tiannan Guo[1,2,3,4]*, Kiryl D. Piatkevich[1,2,4]*

[1]Research Center for Industries of the Future, School of Life Sciences, Westlake University, 600 Dunyu Road, Hangzhou, Zhejiang, 310030, China;

[2]Westlake Laboratory of Life Sciences and Biomedicine, 18 Shilongshan Road, Hangzhou 310024, Zhejiang, China;

[3]Key Laboratory of Structural Biology of Zhejiang Province, Westlake University, 18 Shilongshan Road, Hangzhou 310024, Zhejiang, China;

[4]Institute of Basic Medical Sciences, Westlake Institute for Advanced Study, 18 Shilongshan Road, Hangzhou 310024, Zhejiang, China;

[5]College of Pharmaceutical Sciences, Zhejiang University, 866 Yuhangtang Road, Hangzhou 310024, Zhejiang, China;

[6]Whiting School of Engineering, Department of Biomedical Engineering, Johns Hopkins University, Baltimore, MD 21218, USA;

[7]Department of Biochemistry & Molecular Medical Center, Zhejiang University School of Medicine, Hangzhou, 310058, China;

[8]Research Center for Clinical Pharmacy & Key Laboratory for Drug Evaluation and Clinical Research of Zhejiang Province, The First Affiliated Hospital, Zhejiang University School of Medicine, Hangzhou, 310003, China;

[9]Zhejiang Laboratory for Systems & Precision Medicine, Zhejiang University Medical Center, 1369 West Wenyi Road, Hangzhou, 311121, China.

[#]These authors contributed equally

*Corresponding author(s): Tiannan Guo (guotiannan@westlake.edu.cn); Kiryl D. Piatkevich (kiryl.piatkevich@westlake.edu.cn)

**Supplementary Note 1. Development and optimization of the ProteomEx protocol.** We sought to employ physical magnification of tissue as a new approach for spatially resolved proteomics. Tissue expansion was already realized for super-resolution imaging of biological specimens in the method called Expansion Microscopy (ExM)[1,2]. Physical magnification is achieved via *in situ* synthesis of hydrogel polymer within sample, which can be isotropically expanded in water after mechanical homogenization of the sample to eliminate intermolecular interactions. In the most widely adopted modification of ExM, called protein-retention ExM[3–5], before hydrogel embedding chemically fixed biological sample is treated with chemical anchors, which facilitate incorporation of proteins into polymer chains during *in situ* polymerization. Thus, in the expanded sample proteins remained covalently anchored to polymer mesh. We suggested that anchored proteins can be recovered from the expanded samples in the form of peptides, which can be subjected to MS-based analysis. Therefore, we decided to extend protein-retention ExM workflow with sample microdissection and peptide extraction (**Figure 1A**). To adopt tissue expansion via hydrogel embedding for MS-based analysis, we developed and optimized key steps of the proposed workflow including i) development of a novel hydrogel with enhanced expansion factor and mechanical stability; ii) development of reversible protein anchoring to polymer network; iii) optimization of tissue-hydrogel composite homogenization; iv) development of hydrogel embedded sample staining with colorimetric dye; v) optimization of in-gel digestion and peptide extraction.

We started with the development of a novel hydrogel composition for tissue expansion. Since we sought to analyze large tissue sections, such as coronal brain sections and whole liver sections, it was crucial to use hydrogel compositions, which would allow for manual handling and microdissection of expanded samples without risk of cracks or fracturing. At the same time, we sought to achieve higher resolution by enabling a higher expansion factor. The most widely used hydrogels for ExM are characterized by a linear expansion factor of <4. Higher expansion factors of the acrylate-based hydrogels used in expansion microscopy (ExM) can be achieved either by reducing the concentration of crosslinkers[1,6,7] or by gel re-embedding for iterative expansion[8,9]. However, reduction of crosslinker concentration results in more fragile gels that may not show uniform expansion. Gel re-embedding is not practical as it extends the timeline and adds complexity to the procedure as well as reduces protein retention efficiency. Alternatively, a new gel composition, utilizing N,N-dimethylacrylamide (DMAA) and sodium acrylate (SA) as monomers, can provide up to a 10-fold expansion factor without a need for a crosslinker due to side chain reaction of DMAA molecules. Although the DMAA-based hydrogel was used for protein-retention ExM, it is very soft, thus prone to deformation under gravity force, and requires oxygen-free solution for polymerization reaction. To develop a hydrogel with a high expansion factor and appropriate mechanical stability, we decided to systematically screen for novel hydrogel composition and polymerization conditions assessing expansion factor and mechanical stability. We chose to optimize the DMAA-based hydrogels by screening a large diversity of hydrogel recipes using various comonomers and crosslinkers. As comonomers we used sodium acrylate (SA), sodium methacrylate

(SMA), itaconic acid (IA), trans-aconitic acid (TAA), sodium 4-hydroxy-2-methylenebutanoate (SHMB), ethyl-2-(Hydroxymethyl)-acrylate (EHA), and acrylamide (AA). As crosslinkers we used N,N-Methylenebisacrylamide (MBAA, aka Bis), N,N-dimethylacrylamide (DMAA), Pentaerythritol Tetraacrylate (PT), Trimethylolpropane Propoxylate triacrylate (TPT), Pentaerythritol triacrylate (PA), Dipentaerythritol penta-/hexa-acrylate (DPHA), Trimethylolpropane triacrylate (TTA), Di(trimethylolpropane)-tetraacrylate (DiTA), Trimethylolpropane Trimethacrylate (TTMA), Glycerol propoxylate (1PO/OH) triacrylate (GPT), Trimethylolpropane ethoxylate triacrylate (TET), Pentaerythritol allyl ether (PAE), and N,N-Dimethylaminopropyl acrylamide (DMPAA). We mixed the components in the following molar ratios comonomer:DMAA:crosslinker 1:4:0.01 and tested four different polymerization conditions using the VA-044 initiator at 45ºC, the V-50 initiator at 50ºC, ammonium persulfate at 37ºC, and potassium persulfate at 25ºC. In addition, we explored supplementation of the original ExM hydrogel recipe (SA:AA:MBAA at molar ratio 2.6:1:0.03)[3] with DMAA using SA and SMA as comonomers (SA(SMA):AA:MBAA:DMAA at molar ratio 2.6:1:0.03-0.0005:0.3). Each formed hydrogel was expanded in water to assess expansion factor and sturdiness. First, all formed hydrogels were fully expanded in pure water, placed on the flat surface, and measured for expansion factor calculation and visually inspected for shape integrity. Hydrogels that exhibited cracks or uneven expansion were excluded from further assessment. Hydrogels that passed initial screening were next tested for mechanical stability. Mechanical stability was examined by manual assessment of the fully expanded hydrogels imitating sample handling during ProteomEx workflow (transferring hydrogels from dish to dish, shaking, and dissecting with a scalpel). Hydrogels that did not exhibit cracks or breakage during manual handling were considered mechanically stable. Out of about 400 screened hydrogels, the SMA:DMAA:PAE and SMA:DMAA:TPT compositions exhibited the highest expansion factor while being mechanically stable. To further optimize the selected hydrogel compositions, we assessed expansion factor and mechanical stability at the varied crosslinker concentrations. We found that lowering crosslinker concentration can increase expansion factor without significant reduction of mechanical strength (**Supplementary Table 1**). For further optimization using biological samples, we selected the two hydrogel recipes consisting of SMA:DMAA:PAE in molar ratio 1:4:0.0008 and SMA:DMAA:TPT in molar ratio 1:4:0.0005, which were characterized by maximal linear expansion factors of 8.2 and 8.4, respectively. The linear expansion factor of the new hydrogels was about twice higher than that for the hydrogels traditionally used in ExM[3,5]. We also demonstrated the SMA:DMAA:PAE hydrogel, which was eventually used for MS-analysis of biological tissues, was characterized by the higher or similar stability upon compression compared to conventional ExM hydrogel[3] and DMAA-containing ExM hydrogel (**Supplementary Figure 1**). For example, expanded ExM hydrogel started to fracture at deformation of 49±6% (mean±SD throughout; n = 4 technical replicates), while the ProteomEx hydrogel withstand compression up to 77±5% (n = 2 technical replicates). Stress at fracturing

was similar for all tested hydrogels (18.8±1.7 kPa for ExM, 18.5±5.7 kPa for DMAA-containing ExM, and 19.0±2.2 kPa for ProteomEx).

Next, we quantified the efficiency and quality of peptide recovery from the brain tissue processed with the proposed tissue expansion workflow utilizing two new hydrogel formulas in combination with various chemical anchors and homogenization conditions (see **Supplementary Table 2** for optimization conditions and results). In the conventional ExM protocols, the succinimidyl ester of 6-((acryloyl)amino)hexanoic acid, or AcX for short, is used to modify the primary amine group on proteins with a functional group capable of incorporating into growing polymer chains during *in situ* polymerization.[3,4,10] However, AcX is an expensive and unstable reagent making it impractical for large sample processing. Instead, we decided to explore utility of alternative NHS esters, which can modify protein with acryloyl or allyl groups, such as N-succinimidyl acrylate (NSA) and N-(allyloxycarbonyloxy)-succinimide (NAS), which are characterized by a higher chemical stability during storage and more accessible compared to AcX. We suggested that NHS ester derivatives might be optimal chemical anchors for proteins as they form amide group, which is very stable under neutral pH but can be hydrolyzed at basic pH. This feature may allow reversible protein anchoring, which is important for high-efficiency protein recovery from hydrogels (see **Supplementary Note 3** for more details). Additionally, we chose to test allyl glycidyl ether (AGE), which has an epoxy group for reaction with amine group and allyl group for anchoring to polymer chains. The NSA anchor was previously used for ExM modification, called ZOOM[11], however, NAS and AGE have not been utilized for protein-retention ExM. To perform sample homogenization, an important step to ensure isotropic tissue expansion, we used three different protein denaturation reagents containing urea, SDS, or 2,2,2-trifluoroethanol (TFE). The SDS containing buffers are used for sample homogenization in ExM when epitope retrieval is required for post-homogenization staining[5], however, urea and TFE were not previously used in ExM. We refrained from using non-specific protease ProK, most commonly used in ExM for sample homogenization since it non-specifically digests proteins making downstream peptide recovery and identification less efficient. In-gel digestion of expanded tissue-hydrogel composite for peptide recovery was performed according to the established protocol for protein recovery from PAGE gel for MS analysis including tryptic digestion, reduction, and alkylation steps to improve Cys containing peptides extraction. After initial testing of different chemical anchors, hydrogel compositions, and homogenization buffers we selected the NSA anchor and the SMA:DMAA:PAE hydrogel in combination with SDS containing homogenization buffer for further optimization (**Supplementary Table 2)**. The combination of these reagents provided the highest number of peptide and protein identifications and enabled tissue expansion without defects (**Supplementary Figure 2**).

Next, we optimized the protocol for peptide recovery from tissue-hydrogel composite. As chemical composition of the hydrogel resembles that of polyacrylamide gels used for protein electrophoresis, we decided to adapt the protocol for in-gel digestion of proteins isolated by gel electrophoresis established for MS-based proteomics sample preparation[12,13]. In general, we followed all steps of the in-gel digestion protocol from

*ref.*[12,13] including destaining, hydrogel dehydration, in-gel reduction, and alkylation with some modifications described in the Methods section. In addition, we optimized proteolytic digestion by comparing a combination of trypsin with LysC to only tryptic digestions (**Supplementary Table 3**). Since tryptic digestion yielded higher numbers of peptide and protein identification, we further optimized trypsin concentration confirming that the highest used trypsin concentration (12.5 ng/µL) was optimal (**Supplementary Table 2, 3)**. Before in-gel digestion, we treated the tissue-hydrogel composite with 50 mM Tris buffer at pH=8.8 to facilitate hydrolysis of amide bonds formed by the chemical anchor. To reduce the salt content and improve the purity of the recovered peptides, we replaced NaCl in the homogenization buffer with boric acid and added extra wash steps with methanol, respectively. Altogether these optimizations increased the number of peptide identifications by about 20% from ~25,000 to ~30,000 (**Figure 2C**) and therefore were incorporated into the final ProteomEx protocol, which is described in detail in Methods.

**Supplementary Note 2. Optimization of protein-retention ExM-based protocol for MS analysis.**

While we were finalizing this study, Drelich *et al.* published a conceptually similar method that also relies on the physical magnification of biological tissue to increase the lateral resolution of sampling using manual dissection[14]. The method for tissue expansion utilized by Drelich *et al.* is identical to protein-retention ExM (proExM) developed by Tillberg et al.[3] Therefore, we performed a side-by-side comparison of proExM-based proteomics with the ProteomEx method. To compare ProteomEx with the method described by Drelich et al., we performed tissue expansion under conditions that provided the highest peptide identification number reported in the corresponding paper (see Methods section for a detailed description of the procedure and used reagents). The expansion factor achieved for mouse brain tissue was 2.28±0.04 (n=6 brain slices; **Supplementary Figure 2**). It is also should be noted that expanded tissue was hard to visualize since no staining was performed and only outer boundaries of the tissue were recognized. Initially, for peptide extraction, we followed the protocol described by Drelich et al. More specifically, dissected gel samples were treated with excess 45 mM DTT solution in 50 mM ABB (final concentrations) for 15 min at 50°C followed by alkylation with 100 mM IAA in 50 mM ABB (final concentrations) for 15 min at 22ºC in the dark. Before in-gel digestion, the buffer was removed and an excess of 20 µg/ml trypsin in 50 mM ABB solution was added for ~14h at 37°C in a sealed tube. Digestion was stopped by adding TFA to 1% of the final volume and the solution was collected for further desalting using SOLA-96 well column (ThermoFisher, USA). Subsequent analysis revealed that peptide extraction yield was 15.0±2.7 µg peptides/mg tissues (n=4 samples), which was almost 5-fold lower than that for ProteomEx. Furthermore, under identical analysis conditions (processing ~200 ng of peptides from each sample using a timsTOF Pro mass spectrometer in data-dependent acquisition (DDA) mode), the peptides and proteins identification obtained by the ProteomEx (~30,600 peptides and ~3800 proteins, n=4 samples) was also significantly higher than that provided by the *Anal Chem* (~180 peptides and ~120 proteins, n=4 samples). We

suggested that the low number of identified peptides and proteins was due to inefficient passive diffusion of peptide from the expanded hydrogel. For ProteomEx, the hydrogels were intensively washed and dehydrated before trypsin treatment. Gel dehydration and additional wash steps used for in-gel digestion are important for improved peptide extraction as previously suggested[12,13], therefore we applied peptide recovery procedure we established for ProteomEx to proExM gels. As a results, peptide yield and identification were significantly improved reaching values close to that for ProteomEx (see **Figure 2** and Results section for details). For convenience, we refer to the combination of proExM with our optimized in-gel digestion protocol as proExM-MS.

**Supplementary Note 3. Chemical and post-translational modifications of peptides obtained with ProteomEx.**

The ProteomEx workflow involves protein anchoring and embedding into hydrogel polymer chains via covalent bonds followed by physicochemical treatment with an SDS-containing buffer. Therefore, it is important to verify the chemical modifications that can be potentially introduced during ProtoemEx procedure as well as post-translational modifications recovery.

First, we assessed reversible anchoring of proteins into a polymer network. The NSA and AcX anchors should primarily modify primary amine groups of the amino acid side chains, such as Lys (K), Asn (N), Gln (Q), and Arg (R), which would serve as attachment points to the polymer mesh. To investigate detaching peptides from the polymer network, we compared the ratio of peptides containing K, N, Q, and R amino acids extracted from the expanded tissue with that for samples prepared using in-solution digestion and PCT where chemical modification of amino acids was not used (for this analysis we used raw datasets represented in **Figure 2C**). We observed that the ratios of the peptides containing N, Q, and R were almost identical for all used methods (**Supplementary Figure 5A**). However, the ratios of the peptides containing K were slightly lower for proExM-MS (54.04%) and ProteomEx (56.54%) methods than that for in-solution digestion (63.47%) and PCT (62.51%). For reference, we also compared the ratio of lysine-containing peptides extracted from the expanded samples homogenized either with SDS-contaning buffer or with TFE (for this we used raw datasets represented in **Supplementary Figure 2**). We revealed that the ratio for SDS-based homogenization corresponding to the ProteomEx protocol was 57.3%, which is similar to that for the samples shown in **Figure 2**. However, in the case of TFE-treated samples, the ratio was almost twice lower about 27.5%, which might indicate incomplete retrieval of the K-containing peptides. These results indicated that the protein anchoring in the ProteomEx protocol is reversible and the optimized protocol provides retrieval efficiency of the peptides, that are covalently anchored to the polymer network, comparable with the common sample preparation methods, such as in-solution digestion and PCT.

To further verify the chemical modifications that can be potentially introduced during ProtoemEx procedure as well as post-translational modifications (PTMs) of peptides, we set the variable modifications with anchor mass shift (Mass delta 54.0474 for ProtoemEx corresponding to the modification with NSA anchor, 114.1656 and

168.2130 for proExM-MS corresponding to the modification AcX anchor) on four amino acid sites (K/Q/R/N), which are primary targets of chemical anchor modification. It appeared that all four methods had more than 0.4% peptide fraction with chemical modification corresponding to the NSA anchor (mass delta 54.0474). The macrosamples processed with ProteomEx showed ~1% of peptides with mass delta of 54.0474, which was about twice higher than the fraction of chemically modified peptides obtained with the in-solution digestions and PCT methods (**Supplementary Figure 5B**). However, it should be noted that for the mass delta modification of 54.0474, we found three other different chemical modifications with the matching mass delta that are naturally occurred (see the following link for details http://www.unimod.org/modifications_list.php?a=search&value=1&SearchFor=54.0474&SearchOption=Contains&SearchField=). These naturally occurring chemical modifications may interfere with the real ratio of the ProteomEx anchor modification quantification and cannot be sorted out by analysis. The other analyzed mass shifts did not exceed 0.124% at the peptide level for all analyzed samples (**Supplementary Figure 5B**).

Next, we analyzed the post-translationally and chemically modified peptides for the four methods by MSFragger using open-search mode. We discovered 158 (88.76%) overlapped types of peptide modifications from a total of 178 modifications for all four methods while there were no unique modifications for ProteomEx and five common modifications between in-solution and PCT (**Supplementary Figure 5C**). Furthermore, we conducted a quantitative analysis of peptide modifications to identify their hierarchical clustering. According to the clustering analysis, PCT was similar to the in-solution digestion, followed by ProteomEx. However, results for ProExM-MS were quite different from the other three methods in the quantification of modified peptides (**Supplementary Figure 5D**). These results demonstrated that ProteomEx does not introduce any unique modifications to the peptides compared to in-solution digestion and PCT that can interfere with protein identification.

**Supplementary Table 1.** Screening of hydrogel compositions and their respective expansion factors and mechanic stabilities.

| **Hydrogel composition** | **Molar ratio at 40% w/w concentration** | **Liner expansion factor (fold)[a]** | **Mechanical stability[b]** |
|---|---|---|---|
| SMA+DMAA+PAE | 1:4:0.014 | 6.5 (n=3) | Stable |
| SMA+DMAA+PAE | 1:4:0.012 | 7.04 (n=3) | Stable |
| SMA+DMAA+PAE | 1:4:0.01 | 7.11 (n=3) | Stable |
| SMA+DMAA+PAE | 1:4:0.004 | 7.86 (n=3) | Stable |
| SMA+DMAA+PAE (ProteomEx hydrogel) | 1:4:0.0008 | 8.2 (n=3) | Stable |
| SMA+DMAA+TPT | 1:4:0.03 | 5.3 (n=1) | Stable |
| SMA+DMAA+TPT | 1:4:0.02 | 5.6 (n=2) | Stable |
| SMA+DMAA+TPT | 1:4:0.01 | 6.57 (n=2) | Stable |
| SMA+DMAA+TPT | 1:4:0.0007 | 8.3 (n=4) | Stable |
| SMA+DMAA+TPT | 1:4:0.0005 | 8.4 (n=1) | Stable |

[a]Polymerization conditions: nitrogen environment with 0.2% of the VA-044 initiator for 3 h at 45°C; [b]Mechanical stability was accessed by visually inspected fully expanded hydrogel samples for cracks and breaks after they were manually handled imitating real experiment, *i.e.*, transferred from dish to dish, shaken, rocked, and dissected using a scalpel; values in parentheses correspond to technical replicates.

**Supplementary Table 2.** Development of the ProteomEx workflow and its further optimization.

| Parameters | Aim | Conditions | Results |
|---|---|---|---|
| Homogenization buffer | To find the optimal denatured solutions that are compatible with hydrogel. | 8 M Urea (pH=8.0) | × Hydrogel dissolved |
| | | 0.2 M SDS (pH=7.0) | √ Even tissue expansion |
| | | 50% 2,2,2- Trifluoroethanol (TFE) (v/v) | × Tissue cracked |
| Hydrogel composition, chemical anchor, and homogenization buffer | Establish optimal tissue expansion protocol (see **Supplementary Figure 1** for details) | Hydrogel SMA:DMAA:PAE<br>Anchor NSA<br>Homogenization SDS | √ Average numbers of identified peptides 25,405 and 3,553 proteins |
| | | Hydrogel SMA:DMAA:PAE<br>Anchor NSA<br>Homogenization TFE | × Average numbers of identified peptides 20,857 and 2,844 proteins |
| | | Hydrogel SMA:DMAA:TPT<br>Anchor AGE<br>Homogenization SDS | × Average numbers of identified peptides 10,373 and 2,569 proteins |
| | | Hydrogel SMA:DMAA:TPT<br>Anchor AGE<br>Homogenization TFE | × Average numbers of identified peptides 4,976 and 1,535 proteins |
| | | Hydrogel SMA:DMAA:PAE<br>Anchor NAS<br>Homogenization SDS | × Average numbers of identified peptides 20,914 and 2,889 proteins |
| | | Hydrogel SMA:DMAA:PAE<br>Anchor NAS<br>Homogenization TFE | × Average numbers of identified peptides 11,722 and 1,917 proteins |
| Protease | Optimize proteolytic in-gel digestion (see **Supplementary Table 3** for details) | LysC + Trypsin | × |
| | | Trypsin | √ |
| Concentration of trypsin | To find enzymes that could be adapted to protocol for ProteomEx.<br>Optimized digestion conditions | 0.5 ng/μL | × |
| | | 12.5 ng/μL | √ |
| Coomassie | To facilitate expanded tissue imaging and visualization with the naked eye | No Coomassie staining | × |
| | | Coomassie staining | √ |
| Gel wash solutions | To remove small molecular weight chemicals, such as Coomassie dye, salts, and homogenization reagents | $H_2O$ | × |
| | | 50% methanol (v/v) | √ |
| pH | Adjust pH to facilitate anchor hydrolysis | 50 mM Tris (pH 8.8) | √ |
| | | NaCl (pH 7.0) | × |
| Solutions (gel homogenization) | Minimize the amount of salt introduced into the protocol. | Boric acid | √ |
| | | NaCl | × |
| | | $CaCl_2$ | × |

√ - selected conditions; × - not selected conditions.

**Supplementary Table 3.** Optimization of the proteolytic in-gel digestion.

| **Hydrogel/ chemical anchor** | **Homo-genization reagent** | **Protease concentration[a]** | **Peptide yield (µg)** | **# peptides[b]** | **# proteins[b]** | **Missed cleavages (%)** |
|---|---|---|---|---|---|---|
| SMA:DMAA:PAE/ NSA | SDS | LysC 6.25 ng/µL + Trypsin 12.5 ng/µL | 12.2 | 5723 | 991 | 14.50 |
| SMA:DMAA:PAE/ NSA | SDS | Trypsin 12.5 ng/µL + Trypsin 12.5 ng/µL | 10.3 | 6822 | 1142 | 13.40 |
| SMA:DMAA:PAE/ NSA | SDS | Trypsin 0.5 ng/µL + Trypsin 0.5 ng/µL | 2.2 | 5908 | 1077 | 32.20 |

[a]Proteases were added sequentially in buffer at pH=8.0-8.8 for 4 h and 8 h at 37°C.

[b]MS data were acquired for 1 µg of each sample in DDA mode on Sciex 5600 with 60 min gradient.

**Supplementary Table 4.** Comparison of peptide and protein identifications using different spatially resolved proteomics approaches.

| Method | Tissue | Dimensions (μm) | Volume (nL) | MS Hardware/ Effective LC gradient length for separation | Data acquisition mode/Software/ | Peptides | Proteins | Ref. |
|---|---|---|---|---|---|---|---|---|
| LCM/ nanoPOTs | Mouse liver | 50×50×12 | 0.03 | QExactive Plus MS/97 min | DDA/MaxQuant | 991 | 378 | [15] |
| | | 100×100×12 | 0.12 | | | 3671 | 1039 | |
| | | 200×200×12 | 0.48 | | | 8153 | 1720 | |
| | | 300×300×12 | 1.08 | | | 10022 | 1855 | |
| LCM/ nanoPOTs | Rat brain cortex | 50×50×12 | 0.03 | Orbitrap Fusion Lumos Tribrid MS/115 min | DDA/MaxQuant | 566 | 182 | [16] |
| | | 100×100×12 | 0.12 | | | 2135 | 695 | |
| | | 200×200×12 | 0.48 | | | 6806 | 1828 | |
| LCM/ S-Trap column | Human blood vessel | NR×NR×5 | 6.25 | QExactive HF MS/90 min | DDA/MaxQuant | 1096 | NR | [17] |
| | | NR×NR×5 | 12.5 | | | 1249 | NR | |
| | | NR×NR×5 | 25 | | | 1285 | NR | |
| | | NR×NR×5 | 50 | | | 1369 | NR | |
| | | NR×NR×5 | 100 | | | 1387 | NR | |
| LCM/ IMER | Mouse lung alveoli | NR | 0.194 | QExactive Plus MS/300 min | DDA/MaxQuant | NR | 6 | [18] |
| | | NR | 0.925 | | | NR | 652 | |
| | | NR | 1.85 | | | NR | 1466 | |
| | | NR | 4.62 | | | NR | 1860 | |
| | | NR | 18.5 | | | NR | 2162 | |
| LESA | Rat brain | 1250[a]×12 | 14.72 | QExactive Plus Orbitrap MS/75 min | DDA/Protalizer | 4837 | 860 | [19] |
| | | 1700[a]×12 | 27.23 | | | 7791 | 1119 | |
| | | 2230[a]×12 | 46.9 | | | 8361 | 1175 | |
| | | 2460[a]×12 | 57.0 | | | 8475 | 1165 | |
| DLE | Rat liver | 260[a]×12 | 0.64 | Orbitrap Fusion Tribrid MS/120 min | DDA/ X! Tandem | NR | 671 | [20] |
| | | 357[a]×12 | 1.20 | | | NR | 708 | |
| | | 776[a]×12 | 5.68 | | | NR | 827 | |
| | | 1203[a]×12 | 13.6 | | | NR | 902 | |
| | | 1666[a]×12 | 26.2 | | | NR | 1052 | |
| proExM | Rat brain | 328[a]×12 | 1.29 | Q- Exactive MS/120 min | DDA/MaxQuant | NR | 394 | [14] |
| | | 458[a]×12 | 1.98 | | | NR | 655 | |
| | | 1633[a]×12 | 32.0 | | | NR | 946 | |
| ProteomEx | Mouse brain | 125[a]×30 | 0.37 | timsTOF Pro MS/100 min | PulseDIA/ DIA-NN | 3000 | 1000 | This study |
| | | 162[a]×30 | 0.62 | | | 2986 | 928 | |
| | | 325[a]×30 | 2.48 | | | 15705 | 3044 | |
| | | 487[a]×30 | 5.58 | | | 23898 | 4202 | |
| | | 649[a]×30 | 9.93 | | | 35160 | 5058 | |
| | | 826[a]×30 | 16.1 | | | 37071 | 5105 | |
| | | | | | | | | |

[a]diameter of round specimen from tissue sample; NR – not reported.

**Supplementary Table 5.** Chemical composition of reagents and buffers for ProteomEx.

| Purpose | Solution name | Solution formula | Storage temperature |
|---|---|---|---|
| Fixation | PFA Fixative | 1 mL 10x PBS (final 1×);<br>2.5 mL 16% paraformaldehyde (final 4%);<br>Add water to 10 mL; | Store at RT, avoid air contact, can be stored at –20ºC |
| Disulfide bond reduction | 40x Borate Buffer stock | 3.1 g Boric acid (final 1 M);<br>1.0 g NaOH (final 0.5 M);<br>Add water to 50 mL; | Store at RT |
| | TCEP-HCL stock | 0.5 M<br>5.73 g TCEP-HCL+25 mL dd$H_2O$;<br>Adjust pH to 7.0 with 5 M NaOH;<br>Add dd$H_2O$ to 40 mL; | Aliquots of 1 mL per tube store at –20ºC |
| | BT (reduction buffer with TCEP-HCl) | 40 mL;<br>1 mL 40× Borate Buffer Stock (final 1×);<br>4 mL TCEP-HCl Stock;<br>35 mL dd$H_2O$; | Store at 4ºC |
| Protein anchoring | NSA Stock | 10 mg/mL NSA re-suspended in anhydrous DMSO; | Store desiccated at 4°C |
| | Protein anchoring buffer | 100 mM MES pH 6.0; | Store at 4ºC |
| | Protein anchoring solution | 10 mL;<br>100 µL NSA Stock;<br>9.1 mL Protein anchoring buffer | Freshly prepared |
| | Anchoring termination buffer | 100 mM MOPS pH 7.0 | Store at 4ºC |
| Gelation | PAE stock | 10 mL, (0.115 g/mL, 0.4 M, dissolved in THF);<br>1.15 g PAE;<br>Add THF to 10 mL; | Store at 4ºC |
| | Monomer Solution Stock | 10 mL, pH 6.5;<br>3.137 g N,N-dimethylacrylamide (DMAA) (~3 mL);<br>0.8624 g Sodium methacrylate (SMA);<br>15.4 µL Pentaerythritol allyl ether (PAE stock);<br>dd$H_2O$ 4.635 mL;<br>10% HCl 350 µL | Store at 4ºC |
| | APS stock | 1 mL - standard<br>(10% wt/wt in dd$H_2O$);<br>0.1 g APS;<br>0.9 g dd$H_2O$ | Freshly prepared |
| | TEMED stock | 1 mL - standard<br>(10% wt/wt in dd$H_2O$);<br>0.1 g TEMED;<br>0.9 mL dd$H_2O$ | Store at 4ºC |
| | Activated Monomer Solution (ATMS) | 1 mL - pH 6.5;<br>900 µL Monomer Solution Stock;<br>30 µL APS stock;<br>20 µL TEMED stock;<br>50 µL ddH2O | Freshly prepared |
| Sample homogenization | Homogenization buffer | SDS (500 mL);<br>28.7 g sodium dodecyl sulfate (SDS) 0.2 M;<br>1.545 g boric acid 50 mM;<br>Add dd$H_2O$ to 500 ml;<br>Vortex to mix | Store at RT |

| | | | |
|---|---|---|---|
| | | pH 7.0 | |
| Coomassie blue staining | Coomassie blue staining buffer | 1000 mL;<br>8 ml 125× native Coomassie blue solution;<br>992 mL dd$H_2O$ | Store at RT |
| Gel preprocessing | De-staining buffer | 100 mL;<br>50 mL acetonitrile (ACN);<br>50 mL dd$H_2O$ | Store at RT |
| De-hydration | De-hydration buffer | 100 mL;<br>50 mL ACN;<br>395.3 mg ammonium bicarbonate (ABB) 100 mM;<br>Add dd$H_2O$ to 100 mL; | Store at RT |
| Reduction | TCEP stock solution | 1.5 mL;<br>86.0 mg TCEP 200 mM;<br>Add 100 mM ABB to 1.5 mL; | Aliquots of 0.1 mL per tube store at –20°C |
| | TCEP working solution | 1.5 mL;<br>0.15 mL 200 mM TCEP;<br>Add 100 mM ABB to 1.5 mL; | Freshly prepared |
| Alkylation | IAA stock solution | 1.5 mL;<br>221.95 mg IAA 800 mM;<br>Add 100 mM ABB to 1.5 mL; | Aliquots of 0.1 mL per tube store at –20°C |
| | IAA working solution | 1.5 mL;<br>0.103 mL 800 mM IAA;<br>Add 100 mM ABB to 1.5 mL; | Freshly prepared |
| Enzymatic digestion | Trypsin stock solution | 0.4 mL, 0.25 µg/µL;<br>Add 400 µL 25 mM ABB to 100 µg trypsin; | Aliquots of 0.1 mL per tube store at –20°C |
| | Trypsin working solution | 2 mL, 12.5 ng/µL;<br>100 µL trypsin stock solution;<br>Add 25 mM ABB to 2 mL; | Freshly prepared |
| Peptide extraction | Solvent buffer A | 25 mM ABB solution; | Store at RT |
| | Solvent buffer B | 40 mL;<br>20 mL ACN;<br>1 mL formic acid (FA);<br>Add 19 mL dd$H_2O$ to 40 mL; | Store at RT |
| | Solvent buffer C | 100% ACN; | Store at RT |
| Desalting | Activation buffer | 100% Methanol; | Store at RT |
| | Equilibration buffer A | 10 mL;<br>8 mL ACN;<br>10 µL Trifluoroacetic acid (TFA);<br>Add dd$H_2O$ to 10 mL | Store at RT |
| | Equilibration buffer B/washing buffer | 10 mL;<br>0.2 mL ACN;<br>10 µL TFA;<br>Add dd$H_2O$ to 10 mL; | Store at RT |
| | Elution buffer | 10 mL;<br>4 mL ACN;<br>10 µL TFA;<br>Add dd$H_2O$ to 10 mL; | Store at RT |
| Loading for MS acquisition | MS buffer | 10 mL;<br>0.2 mL ACN;<br>10 µL FA;<br>Add dd$H_2O$ to 10 mL; | Store at 4°C |

**Supplementary Table 6.** Chemicals and reagents used for ProteomEx and their corresponding suppliers and lot numbers.

| | **Chemical Name** | **Supplier** | **Lot Number** |
|---|---|---|---|
| Fixation | 4% Paraformaldehyde (PFA) | Electron Microscopy Sciences | 15710 |
| | PBS | Thermo Fisher | AM9625 |
| Disulfide bond Reduction | Boric acid | Macklin | B802849 |
| | NaOH | Sigma-Aldrich | S817977 |
| | Tris(2-carboxyethyl) phosphine hydrochloride (TCEP-HCl) | Macklin | T819166 |
| Protein Anchoring | N-Succinimidyl Acrylate (NSA) | TCI | S0814 |
| | 6-((acryloyl)amino) hexanoic acid (acryloyl-X or AcX) | Thermo Fisher Scientific | 63392-86-9 |
| | MES buffer | Macklin | M885671 |
| | MOPS buffer | Macklin | M885700 |
| Gelation | Pentaerythritol allyl ether (PAE) | Sigma-Aldrich | 251720 |
| | Tetrahydrofuran (THF) | Macklin | T818769 |
| | N, N-dimethylacrylamide (DMAA) | Sigma-Aldrich | 274135 |
| | Sodium methacrylate (SMA) | Sigma-Aldrich | 408212 |
| | Sodium acrylate | Sigma-Aldrich | 408220 |
| | Acrylamide | Sigma-Aldrich | 146072 |
| | N,N'-Methylenebisacrylamide | Sigma-Aldrich | M7279 |
| | Ammonium persulfate (APS) | Sigma-Aldrich | A3678 |
| | 4-hydroxy-2,2,6,6-tetramethylpiperidin-1-oxyl (4-hydroxy-TEMPO, 97%) | Sigma-Aldrich | 176141 |
| | N, N,N′, N′-Tetramethylethylenediamine (TEMED | Sigma-Aldrich | T7024 |
| Protein Denaturation | SDS (sodium dodecyl sulfate) | Macklin | S817790 |
| Gel washing | Ammonium bicarbonate (ABB) | Sigma-Aldrich | A6141 |
| Gel washing | Acetonitrile (ACN) | Sigma-Aldrich | CLA955-4L |
| Alkylation reaction | Iodoacetamide (IAA) | Sigma-Aldrich | SLBR5819V |
| Lysis | Urea | Sigma-Aldrich | SLBT0537 |
| Lysis | Thiourea | Sigma-Aldrich | T8656 |
| Coomassie blue staining | Commassie Blue Fast Stain Solution | YEASEN | 20309ES03 |

**Supplementary Table 7.** MS settings for PulseDIA

| **Part1** | | | | | | | | | | |
|---|---|---|---|---|---|---|---|---|---|---|
| **ExpType** | **Repetitions** | **KA** | **m1** | **m2** | **CEA** | **KB** | **m3** | **m4** | **CEB** | **Steps** |
| MS1 | 1 | - | - | - | - | - | - | - | - | - |
| PASEF | 1 | 0.703 | 384 | 399 | -1 | 1.119 | 930 | -1 | -1 | 4 |
| PASEF | 1 | 0.71 | 397 | 412 | -1 | 1.126 | 943 | -1 | -1 | 4 |
| PASEF | 1 | 0.717 | 410 | 425 | -1 | 1.133 | 956 | -1 | -1 | 4 |
| PASEF | 1 | 0.724 | 423 | 438 | -1 | 1.14 | 969 | -1 | -1 | 4 |
| PASEF | 1 | 0.731 | 436 | 451 | -1 | 1.147 | 982 | -1 | -1 | 4 |
| PASEF | 1 | 0.738 | 449 | 464 | -1 | 1.154 | 995 | -1 | -1 | 4 |
| PASEF | 1 | 0.745 | 462 | 477 | -1 | 1.161 | 1008 | -1 | -1 | 4 |
| PASEF | 1 | 0.797 | 384 | 399 | -1 | 1.213 | 930 | -1 | -1 | 4 |
| PASEF | 1 | 0.804 | 397 | 412 | -1 | 1.22 | 943 | -1 | -1 | 4 |
| PASEF | 1 | 0.811 | 410 | 425 | -1 | 1.227 | 956 | -1 | -1 | 4 |
| PASEF | 1 | 0.818 | 423 | 438 | -1 | 1.234 | 969 | -1 | -1 | 4 |
| PASEF | 1 | 0.825 | 436 | 451 | -1 | 1.241 | 982 | -1 | -1 | 4 |
| PASEF | 1 | 0.832 | 449 | 464 | -1 | 1.248 | 995 | -1 | -1 | 4 |
| PASEF | 1 | 0.839 | 462 | 477 | -1 | 1.255 | 1008 | -1 | -1 | 4 |
| | | | | | | | | | | |
| **Part2** | | | | | | | | | | |
| **ExpType** | **Repetitions** | **KA** | **m1** | **m2** | **CEA** | **KB** | **m3** | **m4** | **CEB** | **Steps** |
| MS1 | 1 | - | - | - | - | - | - | - | - | - |
| PASEF | 1 | 0.752 | 475 | 490 | -1 | 1.168 | 1021 | -1 | -1 | 4 |
| PASEF | 1 | 0.759 | 488 | 503 | -1 | 1.175 | 1034 | -1 | -1 | 4 |
| PASEF | 1 | 0.766 | 501 | 516 | -1 | 1.182 | 1047 | -1 | -1 | 4 |
| PASEF | 1 | 0.773 | 514 | 529 | -1 | 1.189 | 1060 | -1 | -1 | 4 |
| PASEF | 1 | 0.78 | 527 | 542 | -1 | 1.196 | 1073 | -1 | -1 | 4 |
| PASEF | 1 | 0.787 | 540 | 555 | -1 | 1.203 | 1086 | -1 | -1 | 4 |
| PASEF | 1 | 0.794 | 553 | 568 | -1 | 1.21 | 1099 | -1 | -1 | 4 |
| PASEF | 1 | 0.846 | 475 | 490 | -1 | 1.262 | 1021 | -1 | -1 | 4 |
| PASEF | 1 | 0.853 | 488 | 503 | -1 | 1.269 | 1034 | -1 | -1 | 4 |
| PASEF | 1 | 0.86 | 501 | 516 | -1 | 1.276 | 1047 | -1 | -1 | 4 |
| PASEF | 1 | 0.867 | 514 | 529 | -1 | 1.283 | 1060 | -1 | -1 | 4 |
| PASEF | 1 | 0.874 | 527 | 542 | -1 | 1.29 | 1073 | -1 | -1 | 4 |
| PASEF | 1 | 0.881 | 540 | 555 | -1 | 1.297 | 1086 | -1 | -1 | 4 |
| PASEF | 1 | 0.888 | 553 | 568 | -1 | 1.304 | 1099 | -1 | -1 | 4 |

**Supplementary Figure 1.** Mechanical stability of the ProteomEx hydrogel compared to conventional ExM hydrogel and DMAA-containing ExM hydrogel.

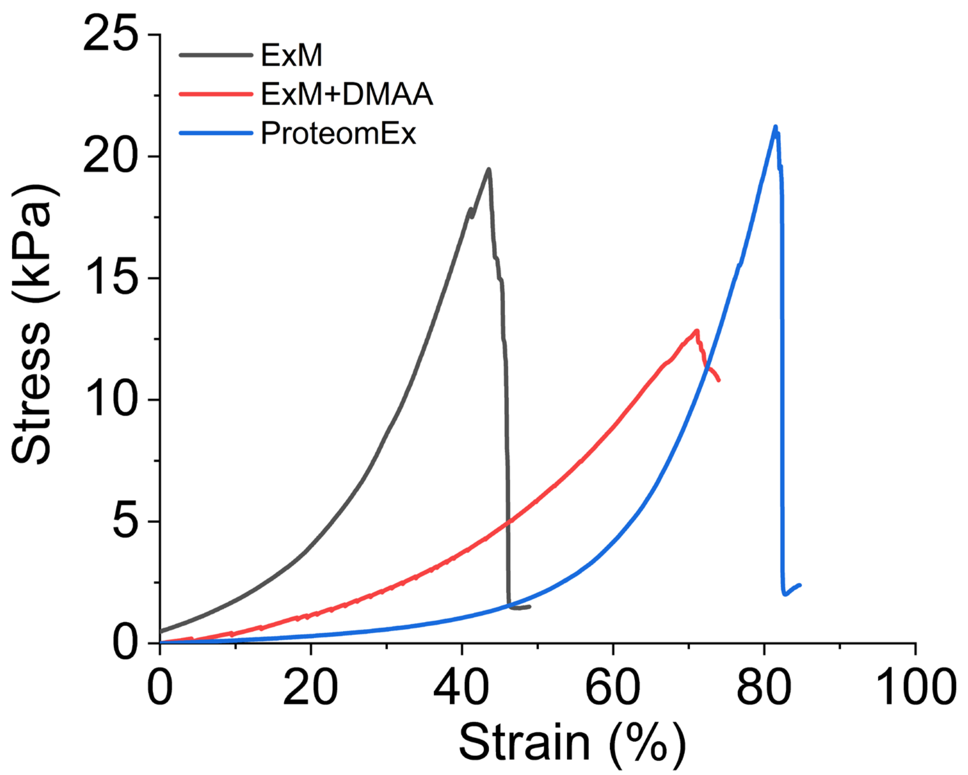


Representative stress–strain curves for expanded ExM (LEF = 4.0), MDAA-containing ExM (LEF = 4.75), and ProteomEx (LEF = 6.25) hydrogels (n = 4, 2, and 2 technical replicates, respectively).

**Supplementary Figure 2.** Optimization of tissue expansion protocol for ProteomEx.

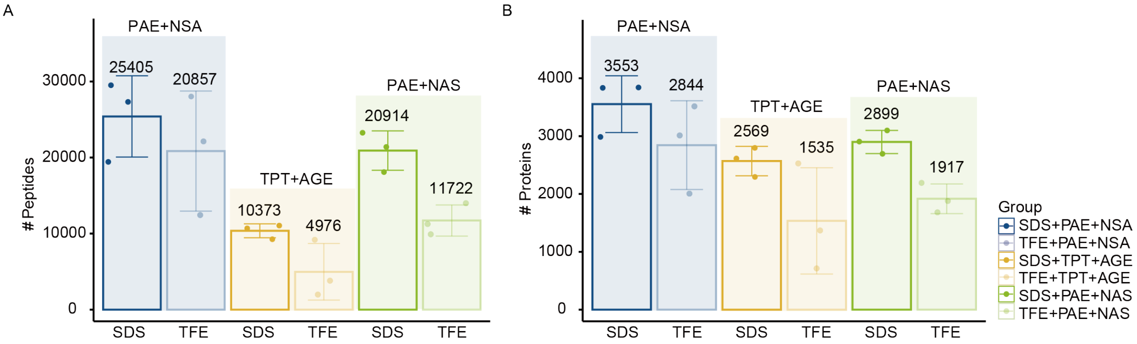


(A) Number of peptide and (B) protein identifications recovered from mouse brain tissue expanded using different hydrogels, chemical anchors, and homogenization buffers (n=3 brain slices from one mouse each; dot, individual data point, bar, mean, whiskers, standard deviation (SD); hydrogels, PAE corresponds to the SMA:DMAA:PAE hydrogel, TPT corresponds to the SMA:DMAA:TPT hydrogel; chemical anchors, NSA, AGE, NAS; homogenization buffer, SDS, TFE). For each sample 200 ng peptides were analyzed using DDA mode on a timsTOF Pro mass spectrometer. Data are presented as mean values ± SD.

**Supplementary Figure 3.** Brain tissue expansion for MS analysis using the proExM protocol.

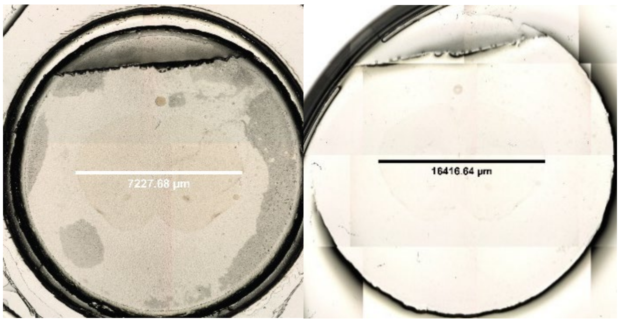


Representative brightfield images of the gelled pre-expansion (left) and expanded (right) mouse brain tissue section expanded using proExM protocol described in *ref*[14] (LEF = 2.28±0.04, n=6 brain slices).

**Supplementary Figure 4.** Peptide quantification and functions of the identified proteins for mouse brain tissue samples processed with in-solution digestion, PCT, proExM-MS, and ProteomEx.

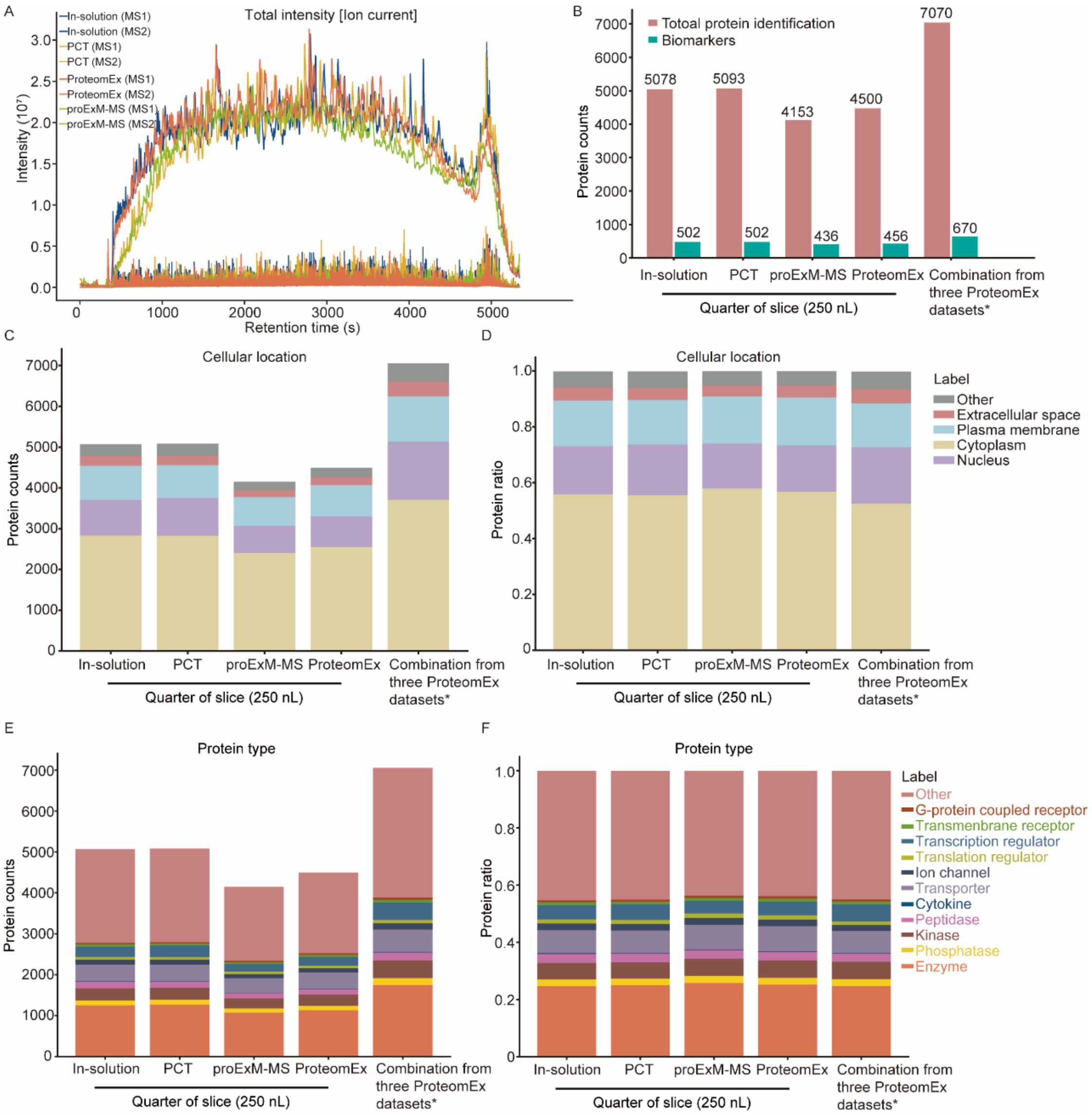


(A) MS1 and MS2 spectra of the peptides for the samples shown Figure 2D on the Bruker timsTOF Pro. Comparison of MS1 and MS2 chromatograms across the LC retention time for the same amounts of peptides (200 ng) from the four methods loaded onto timsTOF (90 min gradient data shown). The spectra of the four methods show that MS1 intensity is around $2.0\times10^7$, the MS2 intensity is around $2.0\times10^6$. (B) The total number of protein identification and biomarkers for the corresponding methods. (C, D) The subcellular locations of (C) the identified protein counts and (D) ratio by the four methods and the ProteomEx combination* for the samples shown Figure 2E. *Combination of three ProteomEx datasets collected from benchmark by DDA, different size gel identification by DIA and AD application by DIA. (E, F) The types of (E) the identified protein counts and (F) ratio by the methods for the samples are shown Figure 2E. Volume in parenthesis corresponds to tissue volumes before expansion.

**Supplementary Figure 5.** Post-translationally and chemically modified peptide analysis for tissue samples processed with four selected methods.

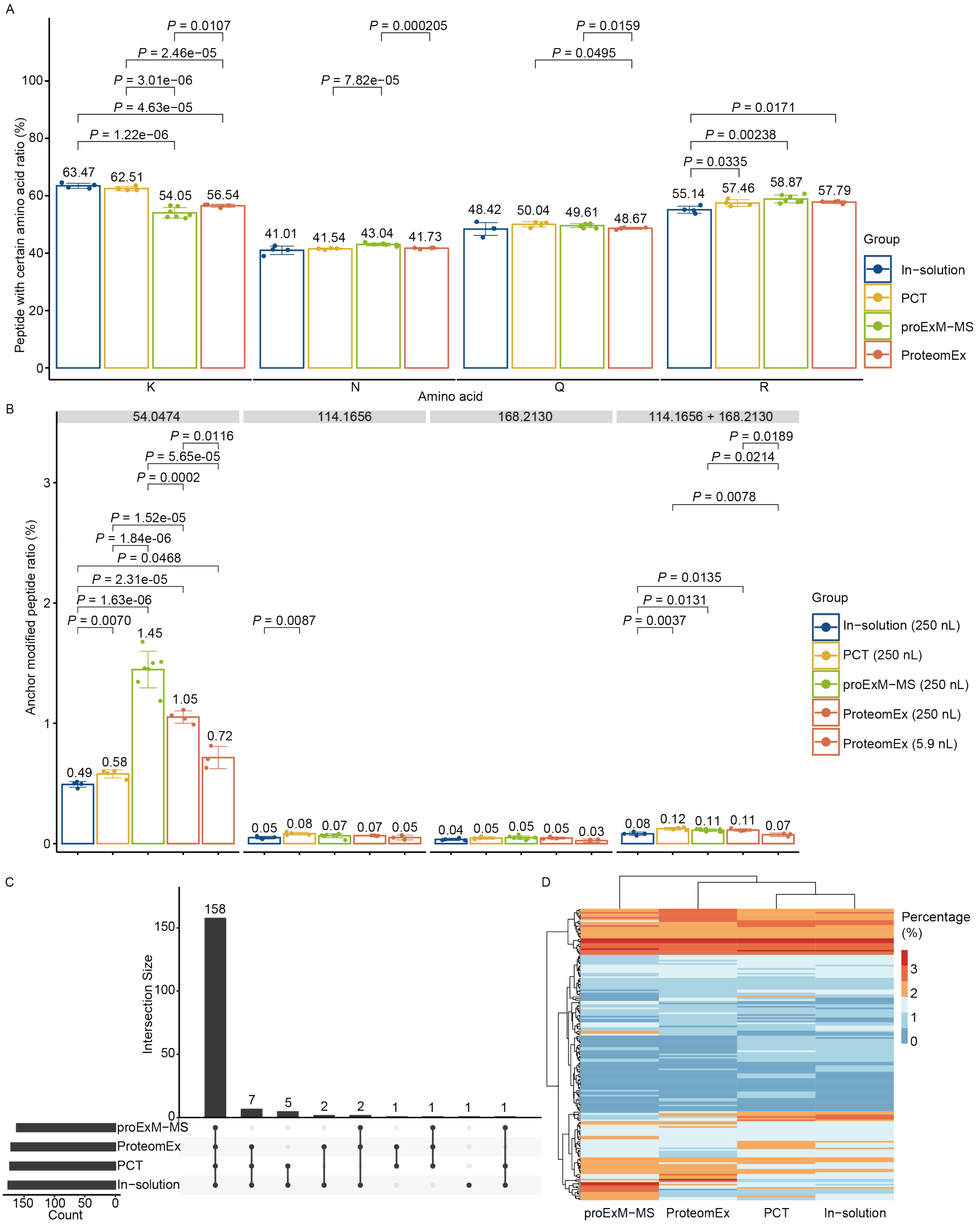


(A) Fraction of identified peptides containing selected amino acids. The peptide with amino acid ratio of the in-solution, PCT, proExM-MS, and ProteomEx methods applied to the mouse brain tissue (n=4, 4, 7, 4-biologically independent samples from one, one, two, and one brain slices, respectively.) Dot, individual data point, bar, mean, whiskers, standard deviation (SD). *P*-values are estimated by Welch's *t*-test (two-sided, pairs without indicated *P*-value are statistically non-significant, i.e., *P*>0.05). Data are presented as mean values ± SD. (B) Anchor-modified peptide analysis. Y-axis indicates the anchor-modified peptide fraction (%). The values on the top of the sections are mass

shift delta, 54.0474 for ProteomEx corresponding to the modification with NSA anchor, 114.1656, and 168.2130 for proExM-MS corresponding to the modification AcX anchor. (n=4, 4, 7, 4 biologically independent samples from one, one, two, and one brain slices, respectively. n=3 punches from one slice from one mouse for ProteomEx (5.9 nL) Dot, individual data point, bar, mean, whiskers, SD. *P*-values are estimated by Welch's *t*-test (two-sided, pairs without indicated *P*-value are statistically non-significant, i.e., $P>0.05$). Data are presented as mean values ± SD.) (C) The Upset plot with the numbers of overlapped modifications of peptides for the four methods. (D) The heatmap illustrating the percentage of different modifications of peptides. Each row represents a type of peptide modification. The rows and columns are clustered by the hierarchical method.

**Supplementary Figure 6.** Reproducibility and stability comparison for the selected sample preparation methods.

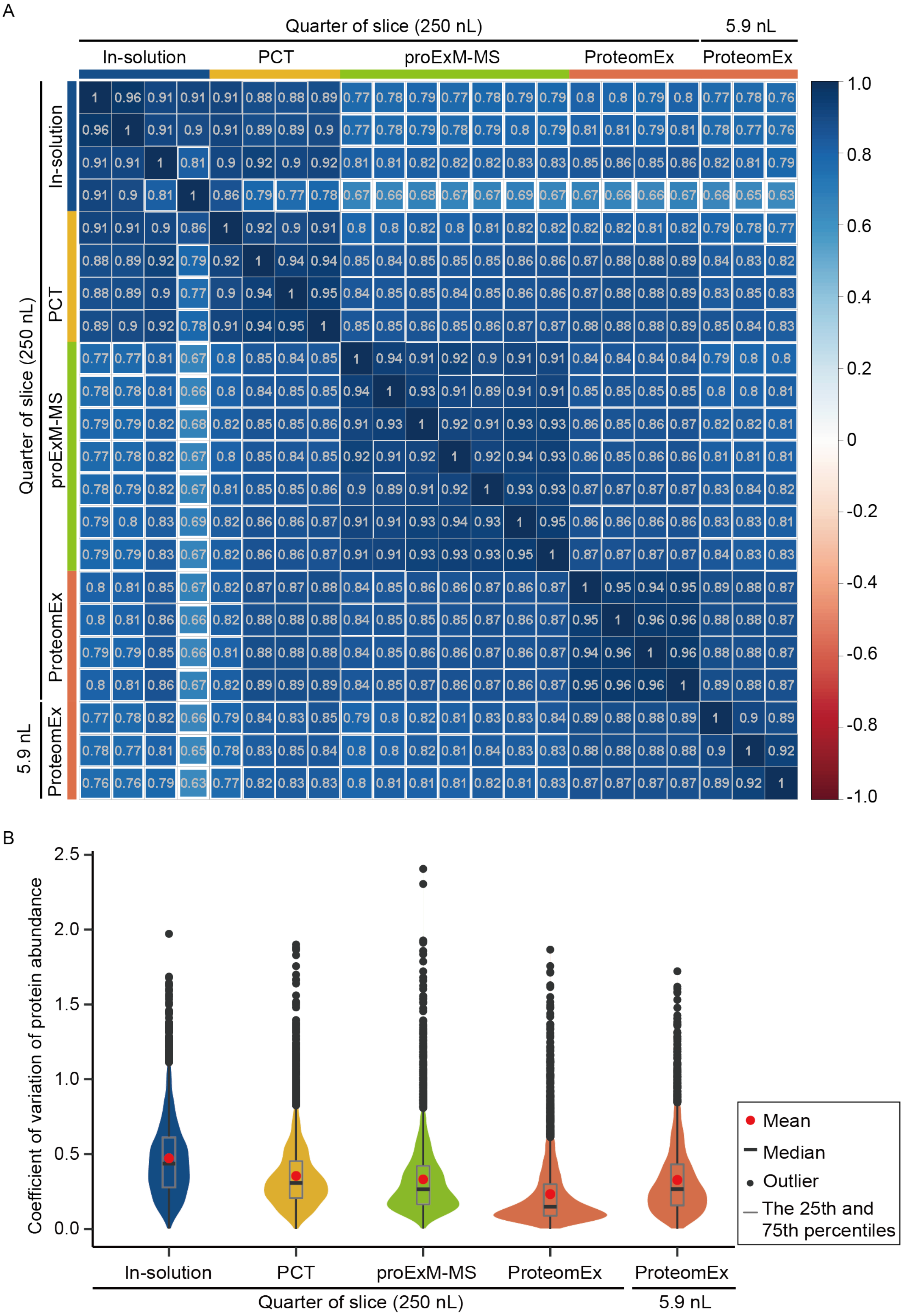


| | In-solution | PCT | proExM-MS | ProteomEx | ProteomEx (5.9 nL) |
|---|---|---|---|---|---|
| 1st quartile | 0.28 | 0.21 | 0.16 | 0.09 | 0.16 |
| Median | 0.44 | 0.31 | 0.26 | 0.15 | 0.27 |
| 3rd quartile | 0.61 | 0.45 | 0.42 | 0.30 | 0.43 |

(A) Heatmap of Pearson correlations for protein quantification for each paired samples from the four sample preparation methods analyzed using the MSFragger software (n=4, 4, 7, 4, 3 biologically independent samples from one, one, two, one and one brain slices for in-solution digestion, PCT, proExM-MS, ProteomEx, and ProteomEx (5.9 nL sample), respectively; the MS raw files corresponding to Figure 2C were used for analysis). The color bar indicates the values of Pearson correlations. (B) Coefficient of variation of quantified protein abundance from the four methods (n= 3919, 4231, 3450, 3777, and 3268 proteins from 4, 4, 7, 4, 3 biologically independent samples from one, one, two, one and one brain slices for in-solution digestion, PCT, proExM-MS, ProteomEx, and ProteomEx (5.9 nL sample), respectively). For each case, the red dot represents the mean; center lines show the medians; box limits indicate the 25th and 75th percentiles; whiskers extend 1.5 times the interquartile range (IQR) from the 25th and 75th percentiles. The black points beyond the end of the whiskers are outlying points. The density curve plotted symmetrically to the left and the right of the box plot is a kernel density estimation to show the distribution shape of the data. Median and IQR are presented at the bottom of the graph.

**Supplementary Figure 7.** Reproducibility and stability of ProteomEx for microsamples.

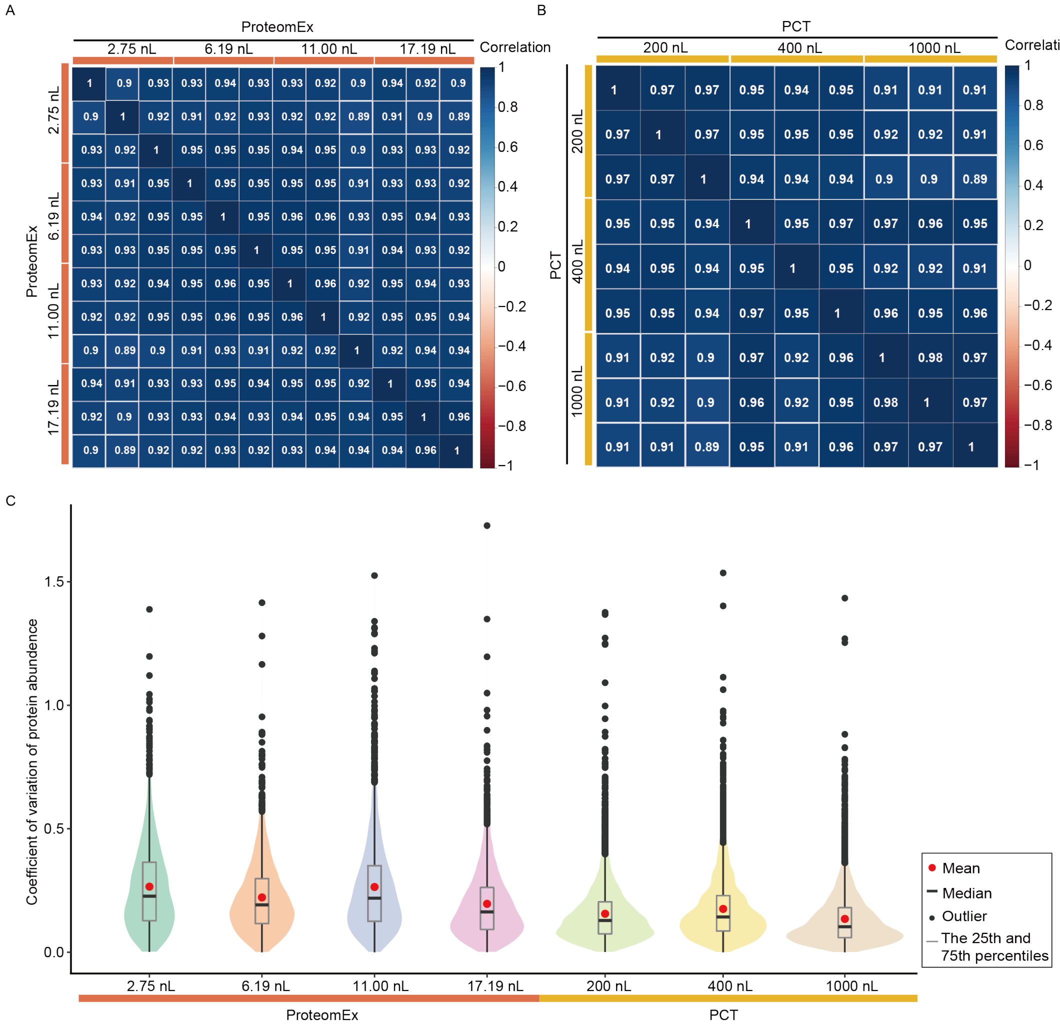


(A, B) Heatmap of Pearson correlations for protein quantification for each sample pair from (A) ProteomEx and (B) PCT analyzed by PulseDIA (the overlapped identified protein ratios in 3 independent runs were 62.9%, 66.7%, 66.7% and 70.3% for the tissue volumes of 2.75 nL, 6.19 nL, 11.00 nL and 17.19 nL, respectively). The color bar indicates the values of Pearson correlations (n=3 adjacent slices from 1 mouse for each method). (C) Coefficient of variation of quantified protein abundance from the two methods (n=1802, 1898, 2202, 2569, 5567, 5816, and 5960 proteins for each sample size from 3 slices from 1 mouse for each method). See **Supplementary Figure 6B** for violin plot description.

**Supplementary Figure 8.** Isotropic analysis of mouse lung tissue sample expansion using ProteomEx.

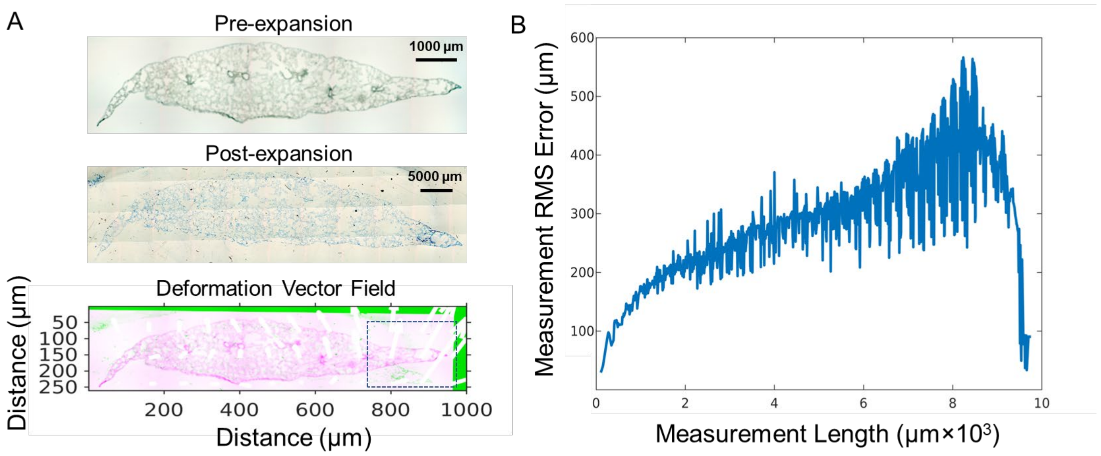


(A) Bright-field images of mouse lung tissue slice pre-expansion (upper panel) and post-expansion (middle panel; Coomassie-stained) and overlay (lower panel) of pre-expansion image (magenta pseudo-color) and registered post-expansion image (green pseudo-color). White arrows represent the deformation vector field (in the overlay images dashed box indicates the area that was displaced during polymerization process and this part of the sample was floating during monomer incubation step; n = 1 slice from 1 mouse). (B) The root-mean-square (RMS) measurement length error for pre- versus post- expansion lung slice images for the experiment shown in A (LEF = 6.4).

**Supplementary Figure 9.** Compatibility of ProteomEx with immunostained and DAPI stained mouse brain tissue.

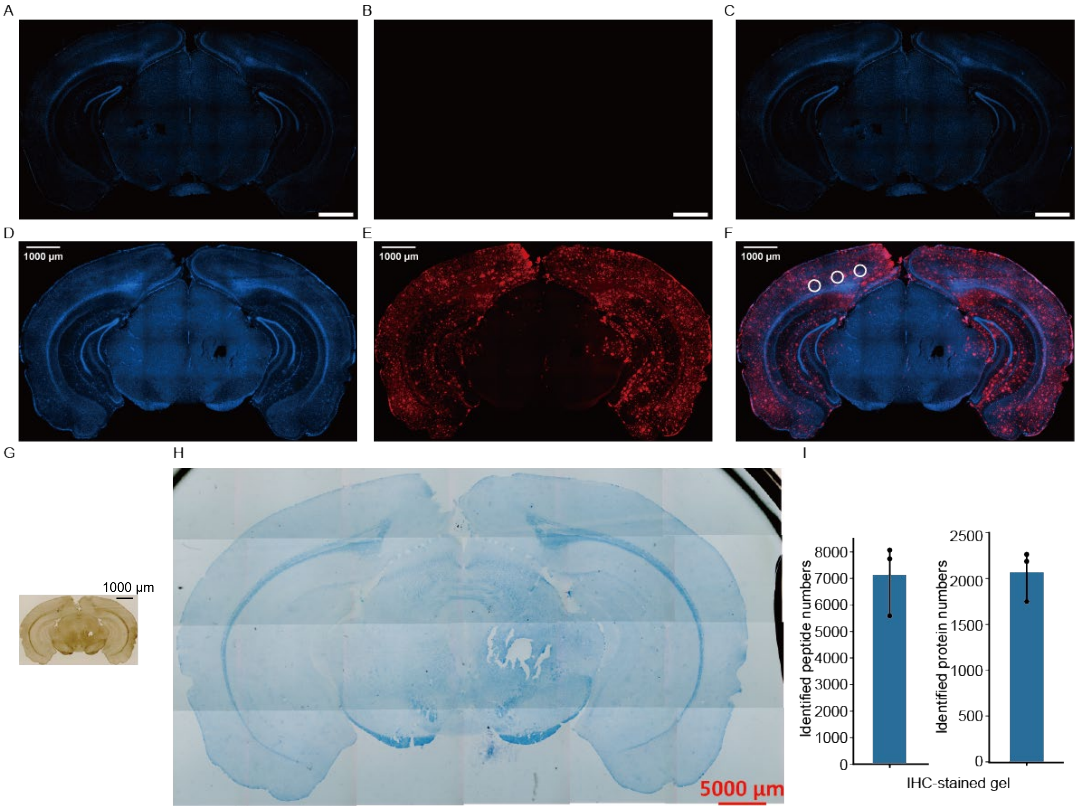


(A, B, C) Representative fluorescence images of mouse brain slice stained with (A) DAPI and (B) anti-β amyloid antibodies and (C) merged image (n= 3 brain slice from 3 wildtype mice). (D, E, F) Representative fluorescence images of mouse brain slice stained with (D) DAPI and (E) anti-β amyloid antibodies and (F) merged image (n= 3 slices from 3 APP/PS1 mice). White circles represented the punched locations used for MS analysis. (G) Representative brightfield images of the pre-expansion and (H) Coomassie-stained expanded mouse brain tissue section (LEF = 6.11-fold; n=3 slices from 1 APP/PS1 mouse). (I) Number of peptide and protein identifications from 2.52 nL brain tissue acquired in by PulseDIA mode (n=3 punches from one mouse brain slice; dot, individual data point, bar, mean, whiskers, standard deviation (SD). Data are presented as mean values ± SD.

**Supplementary Figure 10.** 8-fold brain tissue expansion with ProteomEx.

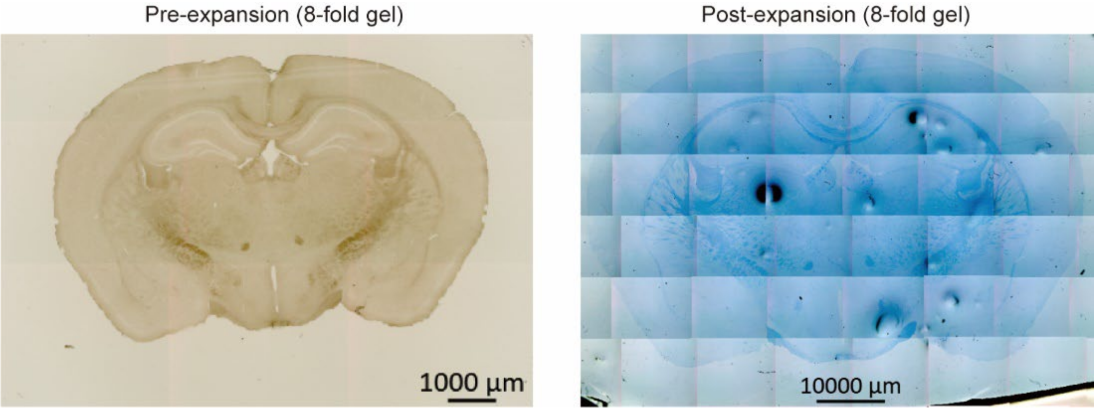


Brightfield images of mouse brain tissue section before expansion (left) and after Coomassie staining (right) and expansion (LEF = 8-fold; n = 1 slice from 1 mouse).

**Supplementary Figure 11.** ProteomEx applied to AD mouse brains.

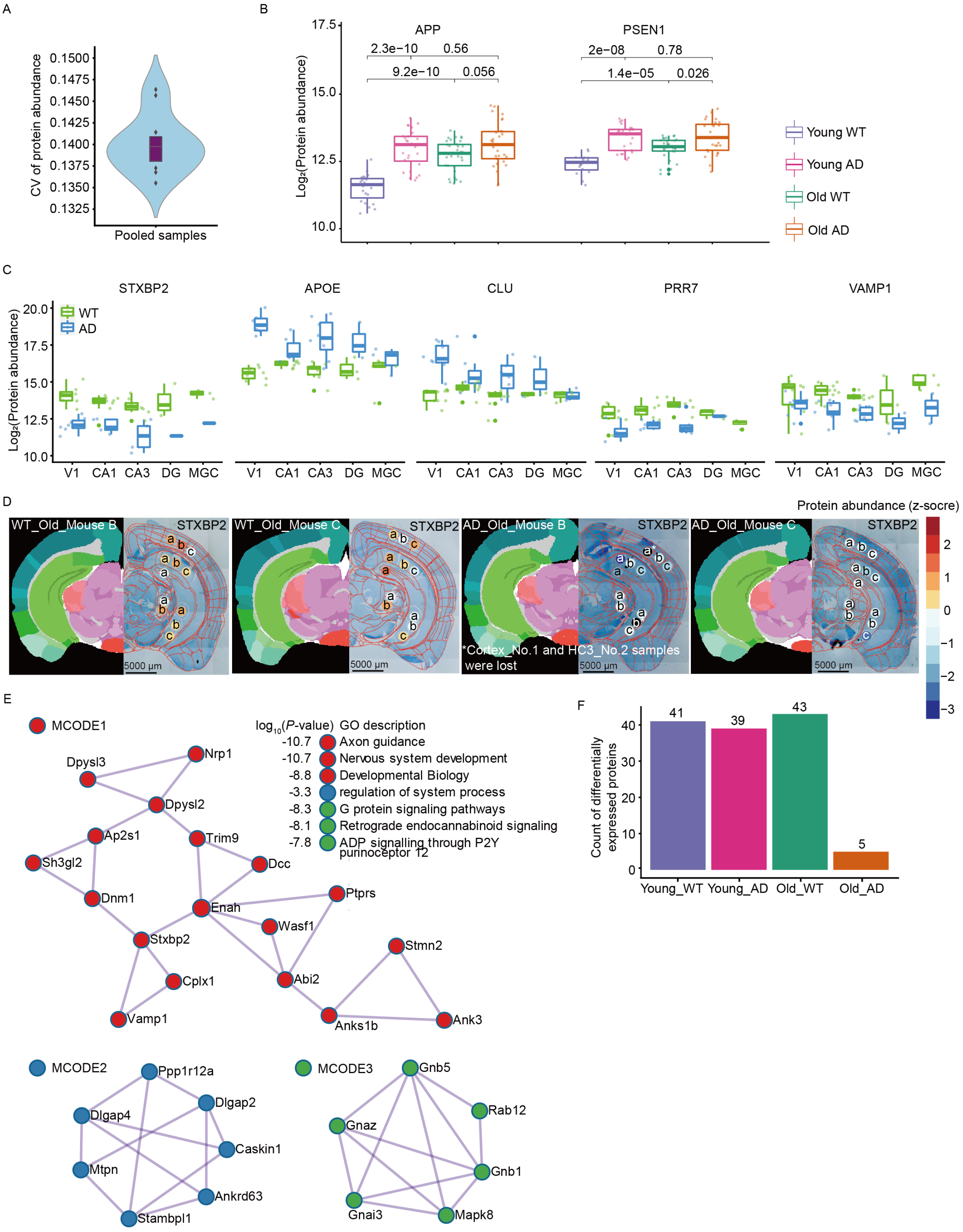


(A) Coefficient variation (CV) of protein abundance for quality control samples prepared as pooled mouse brain peptides (n= 11 technical replicates; see **Supplementary Figure 6B** for violin plot description). (B) Protein expression abundance of APP and PSEN1 in Young WT (n = 30 biologically independent samples from 3 mice), Young AD (n = 28 biologically independent samples from 3 mice), Old WT (n = 35 biologically independent samples from 3 mice), and Old AD groups (n = 29 biologically independent samples from 3 mice; center lines show the medians; box

limits indicate the 25th and 75th percentiles; whiskers extend 1.5 times the interquartile range from the 25th and 75th percentiles, individual data points are represented by dots). (C) Protein expression abundance of STXBP2, APOE, CLU, PRR7, and VAMP1 in different brain regions (V1, CA1, CA3, DG, and MGC) and genotypes (AD and WT) of old mice (n= 17, 16, 17, 6, 8 biologically independent samples for V1, CA1, CA3, DG, MGC from 12 mice. Center lines show the medians; box limits indicate the 25th and 75th percentiles; whiskers extend 1.5 times the interquartile range from the 25th and 75th percentiles, individual data points are represented by dots). (D) Spatial proteomic maps of STXBP2 in biological replicate mouse brain slides. The color bar shows the z-score scaled protein abundance. (E) The top-three significantly enriched clusters of protein-protein interaction for DEPs in CA1 identified by MCODE analysis (Inset, *P* values and gene ontology (GO) description of the presented proteins in the corresponding clusters; colors indicate independent clusters). *P*-values are calculated by hypergeometric test. (F) Count of DEPs (*P* value $<$0.05) among the three subregions of the hippocampus, CA1, CA3 and DG, for each group. *P*-values are calculated by one-way ANOVA.

**Supplementary Figure 12.** Comparison of protein identifications using different spatially resolved proteomics approaches.

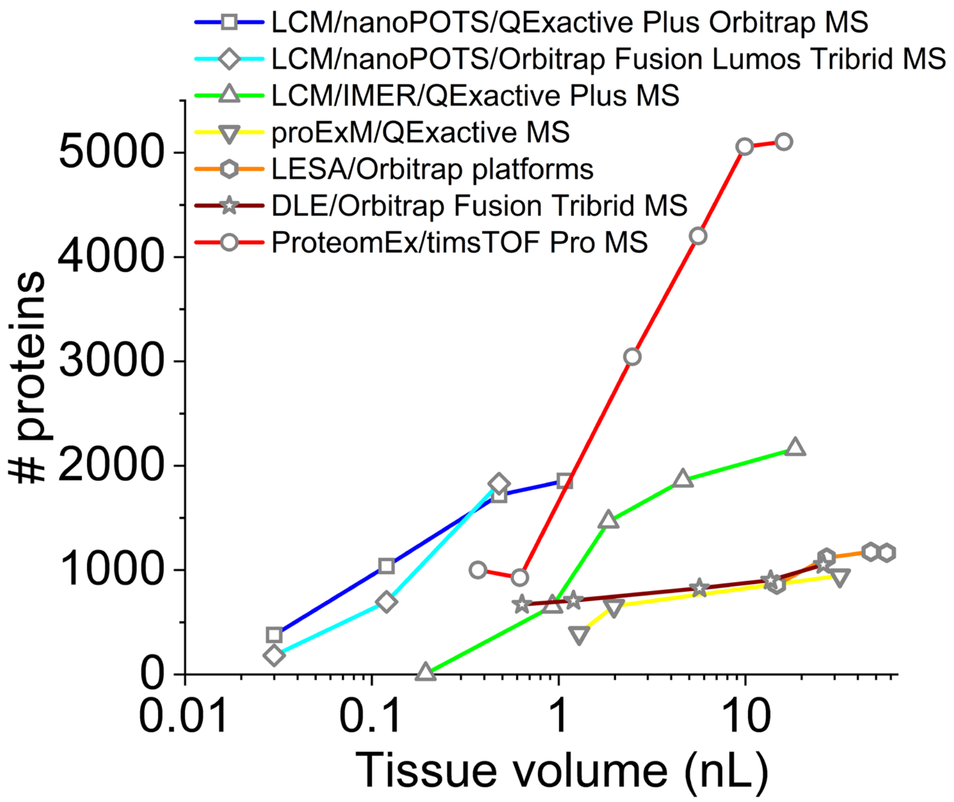


Comparison of protein identifications for subnanoliter and nanoliter tissue volumes achieved with different microsampling and MS techniques for spatially resolved bottom-up proteomics performed on thin (12-30 µm) slices of PFA fixed mammalian tissues. Data for LCM/nanoPOTS/QExactive Plus Orbitrap MS (mouse liver) from *ref.*[15], LCM/nanoPOTS/Orbitrap Fusion Lumos Tribrid MS (rat brain cortex) from *ref.*[21], LCM/IMER/QExactive Plus MS (mouse lungs) from *ref.*[18], proExM/QExactive MS from *ref.*[14], LESA/Orbitrap platforms (rat brain) from *ref.*[19], DLE/Orbitrap Fusion Tribrid MS (rat liver) from *ref.*[20], ProteomEx/timsTOF Pro MS (mouse brain) from this study. LCM, laser capture microdissection; nanoPOTs, nanodroplet processing in one pot for trace samples; IMER, immobilized enzyme reactor; DLE, direct liquid extractions. For details see **Supplementary Table 4**.

**References:**


1. Chen, F., Tillberg, P. W. & Boyden, E. S. Expansion microscopy. *Science (80-. ).* **347**, 543–548 (2015).
2. Wassie, A. T., Zhao, Y. & Boyden, E. S. Expansion microscopy: principles and uses in biological research. *Nat. Methods* **16**, 33–41 (2019).
3. Tillberg, P. W. *et al.* Protein-retention expansion microscopy of cells and tissues labeled using standard fluorescent proteins and antibodies. *Nat. Biotechnol.* **34**, 987–992 (2016).
4. Zhao, Y. *et al.* Nanoscale imaging of clinical specimens using pathology-optimized expansion microscopy. *Nat. Biotechnol.* **35**, 757–764 (2017).
5. Ku, T. *et al.* Multiplexed and scalable super-resolution imaging of three-dimensional protein localization in size-adjustable tissues. *Nat. Biotechnol.* **34**, 973–981 (2016).
6. Okay, O. General Properties of Hydrogels. in *Hydrogel Sensors and Actuators. Springer Series on Chemical Sensors and Biosensors (Methods and Applications)* 1–14 (2009). doi:10.1007/978-3-540-75645-3_1
7. Damstra, H. G. J. *et al.* Visualizing cellular and tissue ultrastructure using Ten-fold Robust Expansion Microscopy (TREx). *Elife* **11**, (2022).
8. Chang, J. B. *et al.* Iterative expansion microscopy. *Nat. Methods* **14**, 593–599 (2017).
9. Gao, R. *et al.* A highly homogeneous polymer composed of tetrahedron-like monomers for high-isotropy expansion microscopy. *Nat. Nanotechnol.* **3**, 12 (2021).
10. Yu, C.-C. (Jay) *et al.* Expansion microscopy of C. elegans. *Elife* 1–78 (2019).
11. Park, H. *et al.* Scalable and Isotropic Expansion of Tissues with Simply Tunable Expansion Ratio. *Adv. Sci.* **6**, 1901673 (2019).
12. Shevchenko, A., Tomas, H., Havliš, J., Olsen, J. V. & Mann, M. In-gel digestion for mass spectrometric characterization of proteins and proteomes. *Nat. Protoc.* **1**, 2856–2860 (2007).
13. Paulo, J. A. Sample preparation for proteomic analysis using a GeLC-MS/MS strategy. *J. Biol. Methods* **3**, e45 (2016).
14. Drelich, L. *et al.* Toward High Spatially Resolved Proteomics Using Expansion Microscopy. *Anal. Chem.* **93**, 12195–12203 (2021).
15. Piehowski, P. D. *et al.* Automated mass spectrometry imaging of over 2000 proteins from tissue sections at 100-μm spatial resolution. *Nat. Commun.* **11**, 1–12 (2020).
16. Zhu, Y. *et al.* Spatially resolved proteome mapping of laser capture microdissected tissue with automated sample transfer to nanodroplets. *Mol. Cell. Proteomics* **17**, 1864–1874 (2018).
17. Herrera, J. A. *et al.* Laser capture microdissection coupled mass spectrometry (LCM-MS) for spatially resolved analysis of formalin-fixed and stained human lung tissues. *Clin. Proteomics* **17**, 1–12 (2020).
18. Clair, G. *et al.* Spatially-Resolved Proteomics: Rapid Quantitative Analysis of Laser Capture Microdissected Alveolar Tissue Samples. *Sci. Rep.* **6**, 1–13 (2016).

19. Ryan, D. J. *et al.* Protein identification in imaging mass spectrometry through spatially targeted liquid micro-extractions. *Rapid Commun. Mass Spectrom.* **32**, 442–450 (2018).
20. Rizzo, D. G., Prentice, B. M., Moore, J. L., Norris, J. L. & Caprioli, R. M. Enhanced Spatially Resolved Proteomics Using On-Tissue Hydrogel-Mediated Protein Digestion. *Anal. Chem.* **89**, 2948–2955 (2017).
21. Zhu, Y. *et al.* Spatially resolved proteome mapping of laser capture microdissected tissue with automated sample transfer to nanodroplets. *Mol. Cell. Proteomics* **17**, 1864–1874 (2018).
