# Supplementary material for: Spatially resolved proteomics via tissue expansion
Source: Nat Commun. 2022 Nov 30;13:7242. doi: 10.1038/s41467-022-34824-2 (PMC9712279; doi:10.1038/s41467-022-34824-2)
Supplement: Supplementary file 2 — Reporting Summary [file 41467_2022_34824_MOESM2_ESM.pdf]

nature research

Corresponding author(s): Tiannan Guo, Kiryl D. Piatkevich

Last updated by author(s): Oct 28, 2022

Reporting Summary

Nature Research wishes to improve the reproducibility of the work that we publish. This form provides structure for consistency and transparency in reporting. For further information on Nature Research policies, see our Editorial Policies and the Editorial Policy Checklist.

Statistics

For all statistical analyses, confirm that the following items are present in the figure legend, table legend, main text, or Methods section.

n/a	Confirmed	
☐	☒	The exact sample size (*n*) for each experimental group/condition, given as a discrete number and unit of measurement
☐	☒	A statement on whether measurements were taken from distinct samples or whether the same sample was measured repeatedly
☐	☒	The statistical test(s) used AND whether they are one- or two-sided<br>*Only common tests should be described solely by name; describe more complex techniques in the Methods section.*
☐	☒	A description of all covariates tested
☒	☐	A description of any assumptions or corrections, such as tests of normality and adjustment for multiple comparisons
☐	☒	A full description of the statistical parameters including central tendency (e.g. means) or other basic estimates (e.g. regression coefficient) AND variation (e.g. standard deviation) or associated estimates of uncertainty (e.g. confidence intervals)
☐	☒	For null hypothesis testing, the test statistic (e.g. *F*, *t*, *r*) with confidence intervals, effect sizes, degrees of freedom and *P* value noted<br>*Give P values as exact values whenever suitable.*
☒	☐	For Bayesian analysis, information on the choice of priors and Markov chain Monte Carlo settings
☒	☐	For hierarchical and complex designs, identification of the appropriate level for tests and full reporting of outcomes
☐	☒	Estimates of effect sizes (e.g. Cohen's *d*, Pearson's *r*), indicating how they were calculated

Our web collection on statistics for biologists contains articles on many of the points above.

Software and code

Policy information about availability of computer code

Data collection	Images were acquired using the Zeiss ZEN 3.1 software. MS data was acquired using the mass spectrometers control software Bruker otofControl version 6.2 and HyStar version 5.1.
Data analysis	The images were analyzed with ImageJ (version 1.53f51) and Zeiss ZEN 3.1 software. Brain tissue annotations were performed using the DeepSlice online app (https://www.deepslice.com.au/) and the QuickNII VisuAlign online app (https://ebrains.eu/service/quicknii-and-visualign/). MS data were analyzed using FragPipe platform (version 15.0) with the MSFragger (version 3.0) and the DIA-NN software (version 1.7.15). Statistical charts are drawn with PRISM (version 9.2.0), R script (version 4.0.3), python (version 3.7) and MATLAB (version R2021a). Isotropic analysis calculation performed with MATLAB (version R2021a). DEPs were calculated by the limma R package (version 3.46.0) with a threshold of Benjamini, Hochberg adjusted P-value <0.05. GO term enrichment was conducted by Metascape web server (http://metascape.org/) using default parameters. Pathway enrichment was performed by IPA, in which the P-value was estimated by right-tailed Fisher’s exact test. The biological process and protein-protein interaction for the top-three significantly enriched clusters were analyzed by MCODE algorithm in the METASCAPE web server. Code is available under the BSD-2-Clause license on GitHub (https://github.com/lilulu777/ProteomEx) and Zenodo [https://doi.org/10.5281/zenodo.7266442].

For manuscripts utilizing custom algorithms or software that are central to the research but not yet described in published literature, software must be made available to editors and reviewers. We strongly encourage code deposition in a community repository (e.g. GitHub). See the Nature Research guidelines for submitting code & software for further information.

Data

Policy information about availability of data

All manuscripts must include a data availability statement. This statement should provide the following information, where applicable:

- Accession codes, unique identifiers, or web links for publicly available datasets
- A list of figures that have associated raw data
- A description of any restrictions on data availability

The mass spectrometry proteomics data generated in this study have been deposited to the iProX database with the dataset identifier IPX0003949000. Raw data including raw images essential to the work are available online as Source Data file and provided in the Supplementary Information. The complete datasets for Figure 3A, B, Figure 4E, Supplementary Figure 11D including raw images are available at FigShare [https://doi.org/10.6084/m9.figshare.21431157] and Zenodo [https://doi.org/10.5281/zenodo.7266442]

Field-specific reporting

Please select the one below that is the best fit for your research. If you are not sure, read the appropriate sections before making your selection.

☒ Life sciences ☐ Behavioural & social sciences ☐ Ecological, evolutionary & environmental sciences

For a reference copy of the document with all sections, see nature.com/documents/nr-reporting-summary-flat.pdf

Life sciences study design

All studies must disclose on these points even when the disclosure is negative.

Sample size	Sample sizes for each experiment are described in the corresponding figure legends. We did not perform a power analysis for sample number, since our goal was to develop a new technology; as noted in ref.49, and recommended by the NIH, "In experiments based on the success or failure of a desired goal, the number of animals required is difficult to estimate…" As noted in the aforementioned paper, "The number of animals required is usually estimated by experience instead of by any formal statistical calculation, although the procedures will be terminated [when the goal is achieved]." The number of samples used were determined empirically based our past experience in developing biotechnologies.
Data exclusions	Samples from AD and normal brain (Figure 4) with fewer than 1464 protein identifications were excluded from downstream data analysis
Replication	All attempts at replication were successful. Figure 1C,D,E n = 20 brain slices from 16 mice. Figure 2A-F n=4, 4, 7, 4 biologically independent samples from one, one, two, and one brain slices for in-solution, PCT, proExM-MS, and ProteomEx methods, respectively. Figure 2G,H n=4 punches per group from 2 mice for ProteomEx, n=3 tissue dissections per group from 1 mouse for PCT. Figure 3A,B n=3, 3, and 3 tissue slices from one mouse each. Figure 3C,D n=3, 3, and 3 punches for each tissue from one mouse each. Figure 3E n= 3 punches from one slice. Figure 4 n=12 mice performed once.
Randomization	Animal perfusion for all experiments was performed in random order. Peptide extraction and MS data acquisition for Figure 4 were conducted in random order to minimize batch effect.
Blinding	The experimenters were not blinded during allocation of animals to each group because we had to make sure that each group contained equal number of animals with matching ages according to the experimental design. For the experiments in Figure 4, experimenters were blinded to tissue samples collection, preparation, and MS data acquisition. Experimenters were blinded during peptide and protein identification step. Experimenters were not blinded during bioinformatic analysis because animal groups had to be compared together.

Reporting for specific materials, systems and methods

We require information from authors about some types of materials, experimental systems and methods used in many studies. Here, indicate whether each material, system or method listed is relevant to your study. If you are not sure if a list item applies to your research, read the appropriate section before selecting a response.

Materials & experimental systems

n/a	Involved in the study	
☐	☒	Antibodies
☒	☐	Eukaryotic cell lines
☒	☐	Palaeontology and archaeology
☐	☒	Animals and other organisms
☒	☐	Human research participants
☒	☐	Clinical data
☒	☐	Dual use research of concern

Methods

n/a	Involved in the study	
☒	☐	ChIP-seq
☒	☐	Flow cytometry
☒	☐	MRI-based neuroimaging

Antibodies

Antibodies used	Primary rabbit monoclonal antibodies for mouse aanti-β amyloid (D54D2) XP® (1:1,000; 8243S; Cell Signaling Technology, US) and secondary antibodies (Alexa Fluor® 647 Goat Anti-Rat IgG H&L, 1:1200; ab150167; Abcam, US) were used in the study.
Validation	All antibodies in the study were used according to the user manuals and validation statements can be found on the respective manufacture website (https://www.cellsignal.com/products/primary-antibodies/b-amyloid-d54d2-xp-rabbit-mab/8243). In the current study the antibodies performed as expected (Supplementary Figure 9).

Animals and other organisms

Policy information about studies involving animals; ARRIVE guidelines recommended for reporting animal research

Laboratory animals	One female MMTV-PyVT transgenic mouse (3-month old) was used for this study as well as three male APP/PS1 (4-month old), three male APP/PS1 (18-month old), three male C57BL/6J (4-month old), and three male C57BL/6J (18-month old).
Wild animals	No wild animals were used in the study.
Field-collected samples	No field collected samples were used in the study.
Ethics oversight	All animal maintenance and experimental procedures were conducted according to the Westlake University Animal care guidelines, and all animal studies were approved by the Institutional Animal Care and Use Committee (IACUC) of Westlake University, Hangzhou, China under animal protocol #19-044-KP.

Note that full information on the approval of the study protocol must also be provided in the manuscript.
